# Analysis of *indoleamine 2*,*3-dioxygenase 1* (*IDO1*) expression of cultured cord blood adherent mononuclear cells as an indicator of atopic risk

**DOI:** 10.1186/1710-1492-10-S1-A72

**Published:** 2014-03-03

**Authors:** Yifei Zhu, Jenny Thiele, Anne K Ellis

**Affiliations:** 1 Department of Biomedical and Molecular Sciences/Medicine, Queen’s University, Kingston, ON, K7L 3N6, Canada

## Background

Maternal atopy is a known risk factor for allergy development in children. This link can be studied to find potential indicators of atopic risk by examining umbilical cord blood. *Indoleamine 2*,*3-dioxygenase 1* (*IDO1*), the initiator of the IDO pathway, plays a regulatory role in the immune response and may differ in expression in the adherent mononuclear cells (AMNC) of atopic and non-atopic individuals. Supernatants of these AMNC cultures may also exhibit different cytokine profiles.

## Methods

Cord blood samples were collected from consenting women undergoing elective Caesarian-sections and atopic status was self-reported. Mononuclear cells were isolated and cryopreserved. Once thawed, AMNCs were cultured and stimulated with interferon-gamma (IFN-γ 1μg/ml or 1ng/ml) with or without control standard endotoxin (CSE 10ng/ml). In each condition, 7.5x10^6^ cells were seeded for gene analysis and 5x10^6^ cells were seeded for cytokine analysis. Cells were lysed for RNA isolation, reverse transcribed and cDNA levels were analyzed using qPCR. Supernatant cytokine levels were analyzed using the Luminex^®^ xMAP^TM^ Technology.

## Results

**Figure 1:**
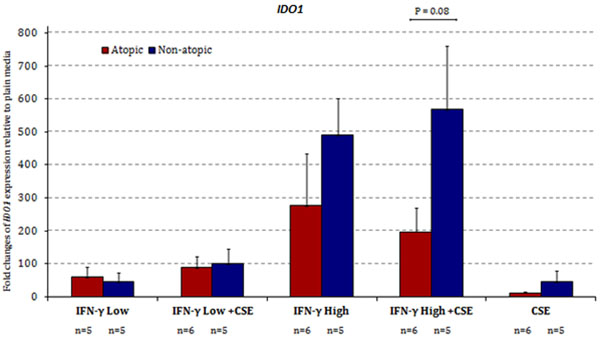
*IDO1* gene expression fold changes relative to plain media control. *IDO1* expression levels were normalized to *HPRT1* expression. The error bars represent the standard error of the mean. Numbers per stimulation group are as indicated beneath the graph. Cultures of atopic and non-atopic AMNCs were plated at 7.5x10^6^ cells per condition. Following 5.5 hours incubation with either plain media, 1 μg/ml IFN-γ, or 1 μg/ml IFN-γ and 10 ng/ml CSE, cells were lysed for RNA extraction. RNA was reverse transcribed and cDNA levels were analyzed.

**Figure 2:**
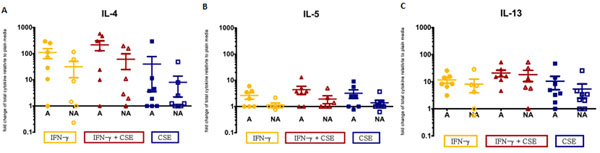
Supernatant cytokine level change relative to plain for Th2 cytokines IL-4 (A), IL-5 (B) and IL-13 (C). Error bars represent the standard error of the mean. Cultures of atopic and non-atopic AMNCs were plated at 5x10^6^ cells per condition. Following 5.5 hours incubation with either plain media, 1 μg/ml IFN-γ, or 1 μg/ml IFN-γ and 10 ng/ml CSE, supernatants were collected and analyzed. A=high atopic risk, NA=low atopic risk. Each condition/atopic risk group contains a minimum of 6 samples.

**Figure 3:**
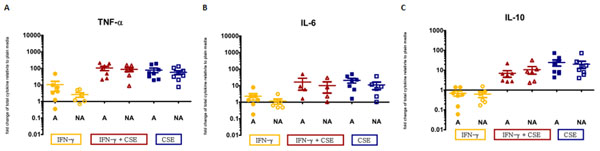
Supernatant cytokine level change relative to plain for pro- and anti-inflammatory cytokines TNF-α (A), IL-6 (B) and IL-10 (C). Error bars represent the standard error of the mean. Cultures of atopic and non-atopic AMNCs were plated at 5x10^6^ cells per condition. Following 5.5 hours incubation with either plain media, 1 μg/ml IFN-γ, or 1 μg/ml IFN-γ and 10 ng/ml CSE, supernatants were collected and analyzed. A=high atopic risk, NA=low atopic risk. Each condition/atopic risk group contains a minimum of 6 samples.

*IDO1* expression was significantly increased in all stimulated conditions (P<0.05) except for the CSE only condition. The high atopic risk group displayed trend towards decreased *IDO1* expression, however, high and low atopic risk groups did not show significant differences (Figure 1). Supernatant cytokine analysis show heightened levels of Th2 cytokines IL-4, IL-5, IL-13 (Figure 2). Similarly, heightened levels of TNF-α and IL-6 were observed, while levels of IL-10 were decreased in the high atopic risk samples in all stimulated conditions (Figure 3).

## Conclusions

Preliminary differences detected suggest that further research could elucidate a suitable biomarker to predict atopic risk. Due to the lack of significant differences between high and low atopic risk groups for *IDO1* expression and cytokine expression, a reliable biomarker was not determined in this study.

